# A Unique Case of Unicornuate Uterus With a Non-communicating Rudimentary Horn and Hematometra

**DOI:** 10.7759/cureus.32640

**Published:** 2022-12-17

**Authors:** Pooja Ladke, Ashish N Ambhore, Avinash Dhok, Kajal Mitra, Vrushali Dalvi

**Affiliations:** 1 Department of Radiodiagnosis, NKP Salve Institute of Medical Sciences (NKPSIMS) and Lata Mangeshkar Hospital (LMH), Nagpur, IND

**Keywords:** magnetic resonance imaging, infertility, mullerian duct anomalies, rudimentary horn, unicornuate uterus

## Abstract

Congenital uterine anomalies are a rare type of malformation involving female genitalia caused by abnormal development of the Mullerian duct system. Patients having an obstructive type of uterine anomalies are very much likely to develop obstetric and gynecological complications usually at the age of menarche or later in the course of life. In this case report, we present a case of a young female patient having severe dysmenorrhea which is caused by obstructive hematometra in the rudimentary horn which is not communicating with the uterine cavity. Ultrasonography (USG) was used to make a differential diagnosis of a probable congenital abnormality, which was subsequently validated by magnetic resonance imaging (MRI), which revealed a uterine cavity having a single cornu on the left side seen to be connecting with the cervix and a dilated rudimentary horn on the right side. The patient underwent the excision of the rudimentary horn laparoscopically. This case emphasizes the importance of identifying patients having anomalies involving the uterus to provide appropriate treatment to the patient and to prevent adverse outcomes for her reproductive potential.

## Introduction

The term unicornuate uterus is used to represent a complex set of Mullerian duct abnormalities. Accurate categorization using hysterosalpingography (HSG), ultrasonography (USG), and magnetic resonance imaging (MRI) requires great attention to the smallest details because each technique has its own strengths and limitations. A unicornuate uterus is caused by the normal development of one Mullerian duct and aberrant development, i.e., hypo- or aplasia of the other [1].

More than one diagnostic imaging modality is frequently used in the proper evaluation, and treatment can be either medical or surgical. Although current imaging methods such as USG and MRI are strongly predictive of Mullerian anomalies, unusual presentations of common disorders must be considered [2].

## Case presentation

Patient information

A 15- year-old unmarried girl presented with a history of severe lower abdominal pain during menstruation for two days which was associated with two episodes of vomiting. On admission, the patient was stable, afebrile, acyanotic, and anicteric, with normal vital signs and physical examination. Her menstrual cycles commenced at the age of 12 years and was having irregular cycles with a duration of 30-40 days and lasting for four to five days. She denied any medical or surgical history or drug allergy.

Clinical findings

**Figure 1:**
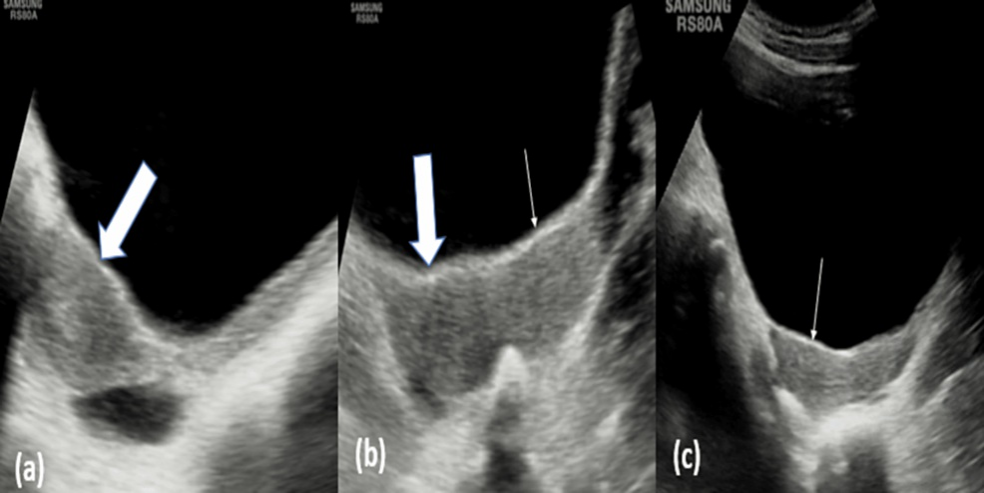
USG pelvis images: (a) axial section showing a non-communicating, blood-filled rudimentary uterine horn (block arrow), (b) the non-communicating, blood-filled rudimentary uterine horn (block arrow) which is next to a mature unicornuate uterus (line arrow), and (c) longitudinal section showing the unicornuate uterine horn (line arrow) in the same patient.

The USG of the pelvis (Figures 1a-1c) revealed an isoechoic structure with echogenic content lying behind the urinary bladder and on the right of a normal uterine body and fundus which appeared to be continuous with one cervix.

Both ovaries were identified separately and were normal. Both kidneys were normal in our patient. No HSG or transvaginal USG study was undertaken as the patient was unmarried. The patient was advised MRI pelvis for further evaluation.

**Figure 2:**
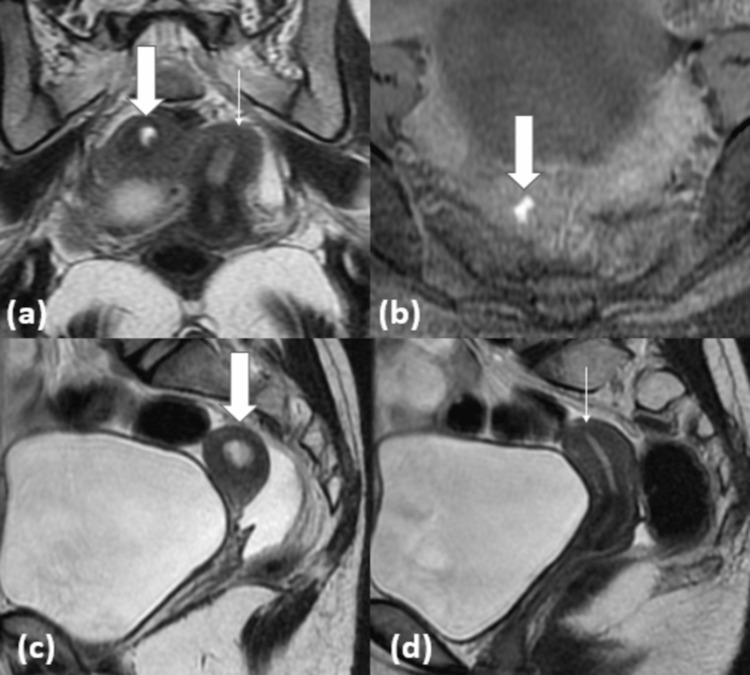
MRI images (a) coronal T2WI showing a non-communicating blood-filled rudimentary horn (block arrow) lying next to a mature unicornuate uterus (line arrow), (b) axial T1 fat-suppressed image showing hyperintense fluid in the horn on right side confirming hemorrhagic content in the obstructed rudimentary horn suggesting hematometra, (c) sagittal T2WI showing blood-filled rudimentary horn (block arrow) not communicating with the cervix, and (d) sagittal T2WI showing normal uterine horn (line arrow) communicating with the cervix.

MRI of the pelvis (Figures 2a-2d) revealed a globular structure measuring approximately 2.3 x 3.1 x 4.5 cm seen lying on the right side of the uterus. The MR morphology of this globular structure was indicative of uterine in origin with a maintained zonal architecture. It had internal components that were hyperintense on T1-weighted images (WI) and T2WI. It did not demonstrate any obvious connection with the normal uterine lumen.

Both ovaries were normal with multiple developing follicles within.

Diagnosis

A diagnosis of the unicornuate uterus which is a congenital anomaly along with a non-communicating functioning rudimentary horn with hematometra in the obstructed element was made.

Therapeutic intervention

**Figure 3:**
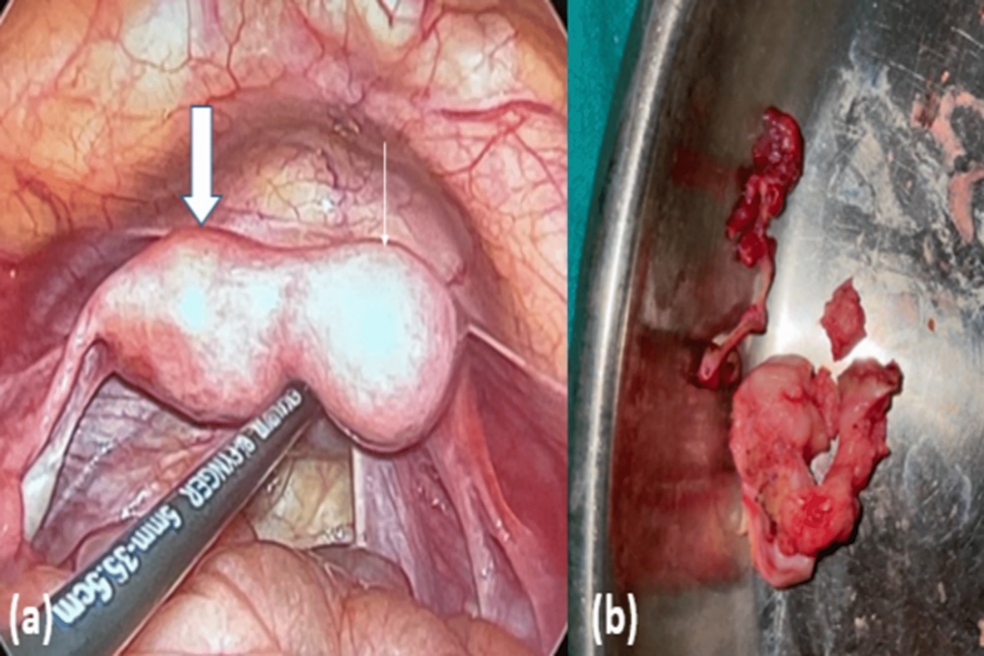
(a) Laparoscopic intraoperative image showing the rudimentary horn (block arrow) adjacent to the unicornuate uterus (line arrow), and (b) post-operative specimen image showing resected rudimentary horn.

The patient underwent laparoscopic resection of the rudimentary horn (Figures 3a, 3b).

## Discussion

Imaging serves to identify, categorize, and direct the surgical management of various uterine anomalies. Due to its excellent accuracy in identifying and properly characterizing Mullerian duct abnormalities, MRI is currently the modality of choice [3].

A rare but frequently treated cause of infertility in women is congenital uterine or Mullerian duct abnormalities. They are predicted to affect 0.1%-0.5% of females. Reproductive issues in women with Mullerian duct malformations include a greater prevalence of infertility, frequent spontaneous abortions in the first trimester, fetal malposition, and preterm labor. Imaging serves to diagnose and identify defects that can be corrected surgically, and the surgical strategy may change depending on the imaging results [4].

Because of the intimate developmental connection between the paramesonephric and mesonephric ducts, renal tract abnormalities are related to Mullerian duct anomalies in up to 30% of cases. Agenesis of the kidneys is the most prevalent renal tract pathology linked with Mullerian duct anomaly [5].

**Figure 4:**
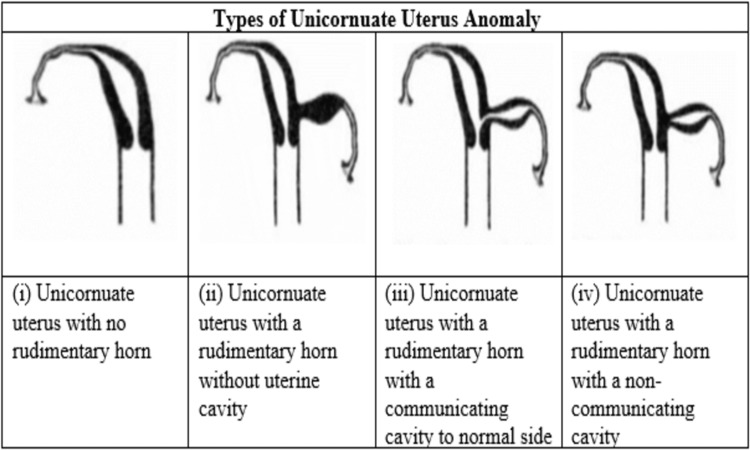
Types of unicornuate uterus anomaly

The functioning endometrium of the rudimentary horn secretes hormones causing hematometra along with an increased incidence of adenomyosis and endometriosis in other parts of the body [6]. Unicornuate uterus anomaly can be of four types (Figure 4) [7].

The absence of an endometrial cavity in a primitive horn poses little risk and is usually not a reason for surgical intervention. Endometrium in the rudimentary horn, on the other hand, is notable and must be documented [8].

## Conclusions

This case report reveals a rare case of a unicornuate uterus with an obstructed contralateral rudimentary horn. Our case falls into class II-B of the American Society for Reproductive Medicine Classification Scheme. The case showed evidence of a unicornuate uterus with an obstructed contralateral rudimentary horn with hematometra. This case was treated with laparoscopic resection of the rudimentary horn. On follow-up, the patient was stable. This case report adds to the knowledge of the significance of radiological imaging in the diagnosis of Mullerian duct anomalies.
